# Supplementary material for: Epigenomic landscape of human colorectal cancer unveils an aberrant core of pan-cancer enhancers orchestrated by YAP/TAZ
Source: Nat Commun. 2021 Apr 20;12:2340. doi: 10.1038/s41467-021-22544-y (PMC8058065; doi:10.1038/s41467-021-22544-y)
Supplement: Supplementary file 1 — Supplementary Information [file 41467_2021_22544_MOESM1_ESM.pdf]

Supplementary Information

Epigenomic landscape of human colorectal cancer unveils an aberrant core of pan-cancer enhancers orchestrated by YAP/TAZ

Della Chiara *et al.*

Supplementary Figures 1 – 6

Supplementary Tables 1 – 3

Supplementary References

Supplementary Figure 1

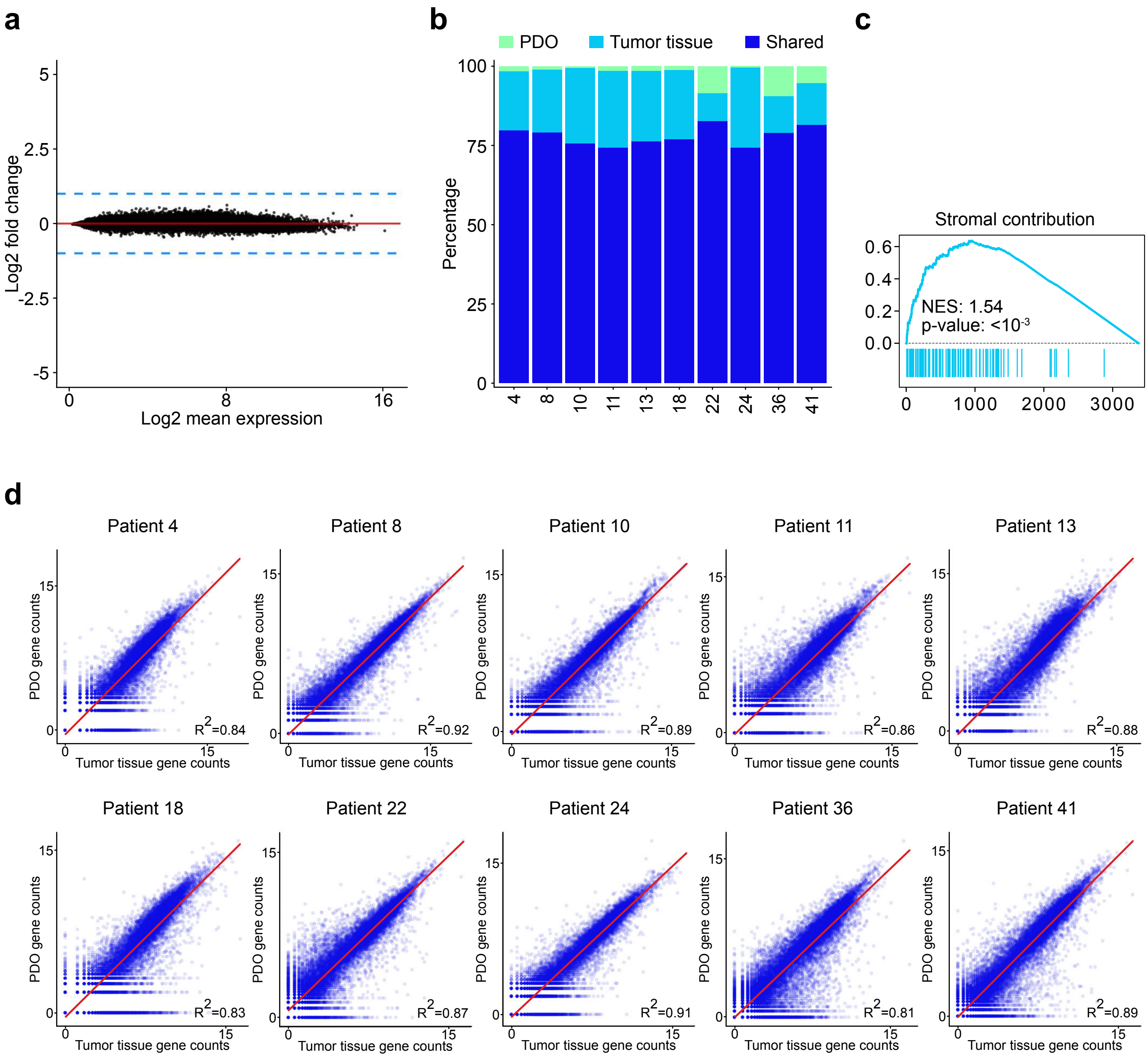

Supplementary Figure 1

PDOs transcriptional stability and gene expression correlation with primary tumors

a, MA plot of log2 mean gene expression over log2 fold-change showing the lack of differentially expressed genes between early and late passages of organoids.

b, Concordance of expressed genes detected in patient-derived organoids (PDOs) and corresponding tumors. Bar graph represents the proportion of expressed genes (gene count > 5) that is shared between each PDO and its corresponding tumor, and those detected only in the PDO or parental tumor.

c, Genes expressed in primary tumors but not in PDOs (Fig. 1e, Venn diagram, n = 3,412) are enriched for gene signatures of stromal cells[1]. GSEA with the normalized enrichment score (NES) and nominal *P*-value using 1000 permutations reported.

d, Pearson correlation of gene expression between matching pairs of tumor tissue and derived PDO.

Supplementary Figure 2

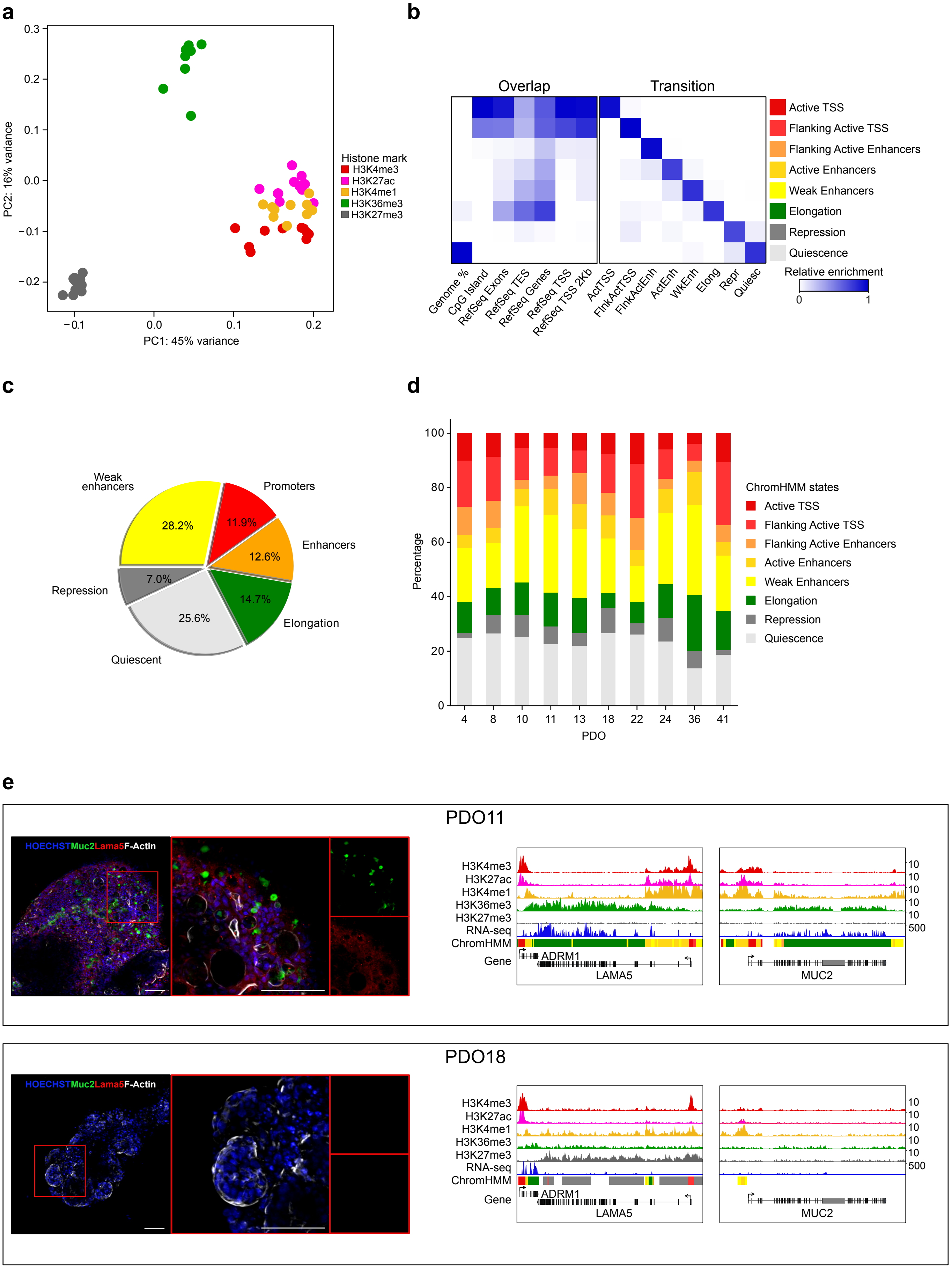

Supplementary Figure 2

Genomic distribution of the *de novo* chromatin states across human CRC PDOs and correlation with 3D immunofluorescence analysis

a, PCA of input normalized ChIP-seq signals for the five histone modifications used to build the ChromHMM 8-state model.

b, Heatmaps showing the annotation of the ChromHMM 8-states with known genomic features (Overlap) and the probability that a state is found in the proximity of another state (Transition).

c, Average proportion of each chromatin state over all PDOs. The chromatin segments for active/flanking TSS and active/flanking enhancer states are merged into the promoter and enhancer functional elements, respectively. TSS, transcription start site.

d, Distribution of the eight ChromHMM states for each PDO.

e, Epigenomic and transcriptional profiles of the *LAMA5* and *MUC2* genomic loci reflect their protein expression levels. Confocal images of 3D immunofluorescence whole-mount analysis on CRC PDOs 11 and 18 stained for MUC2 (green), LAMA5 (red), and F-Actin (white) (left panel). Tracks of histone modification profiles along with RNA-seq signals and ChromHMM states for *LAMA5* and *MUC2* in PDOs 11 and 18, respectively (right panel). The ChromHMM tracks denote regions identified as promoter (red), active enhancer (orange), weak enhancer (yellow), elongation (green), repressed (grey) or quiescent (white) states (Fig. 2c). PDO, patient-derived organoid.

Supplementary Figure 3

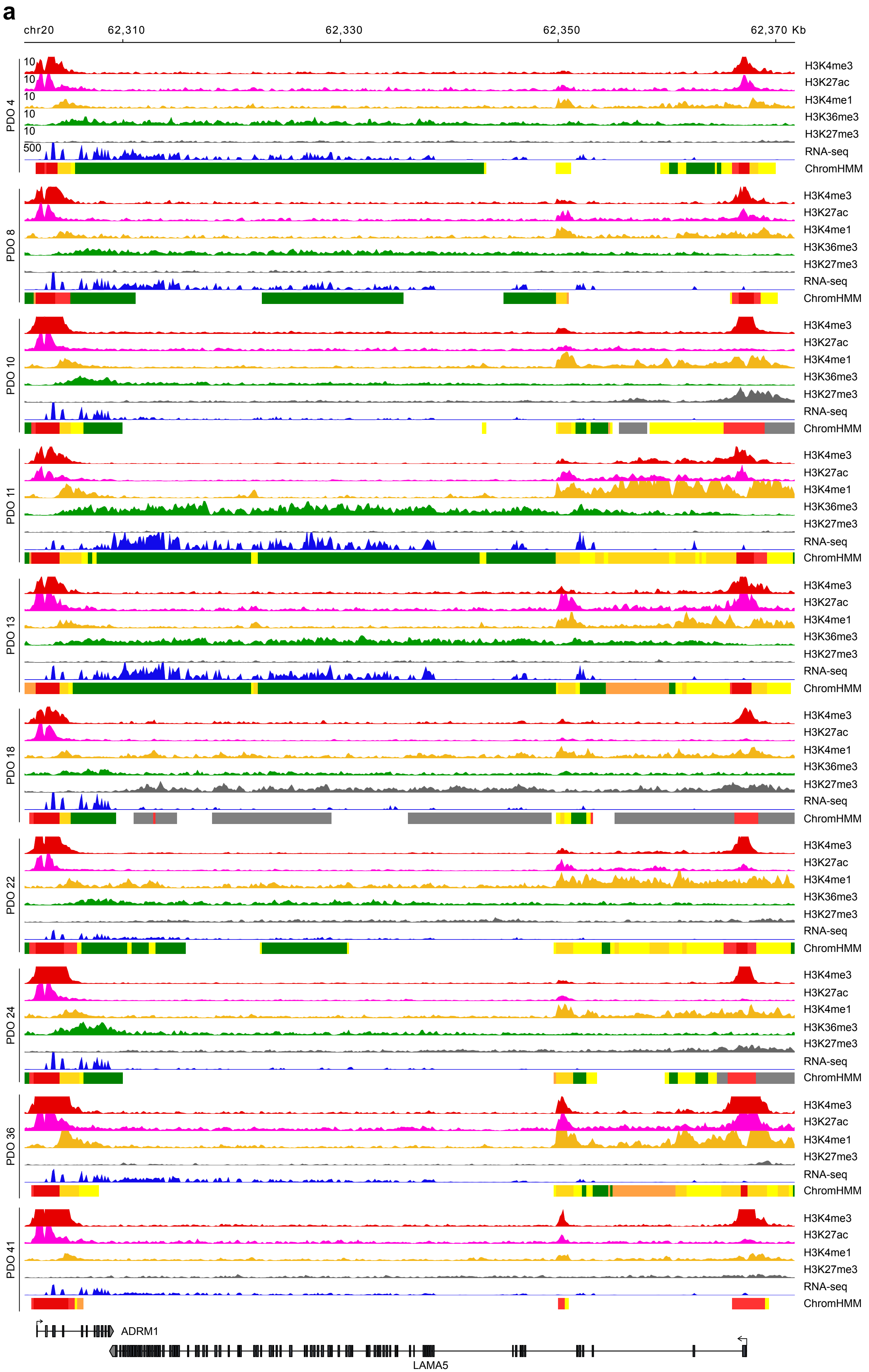

Supplementary Figure 3

Genomic overview of the *LAMA5* genomic region

a, Genomic overview of the *LAMA5* region (hg38, chr20: 62,300,981-62,371,728) for all ten patient-derived organoids (PDOs). The tracks show H3K4me3, H3K27ac, H3K4me1, H3K36me3 and H3K27me3 profiles, RNA-seq signals and ChromHMM states. See Fig. 2c,e and Supplementary Fig. 2e for details on ChromHMM tracks.

Supplementary Figure 4

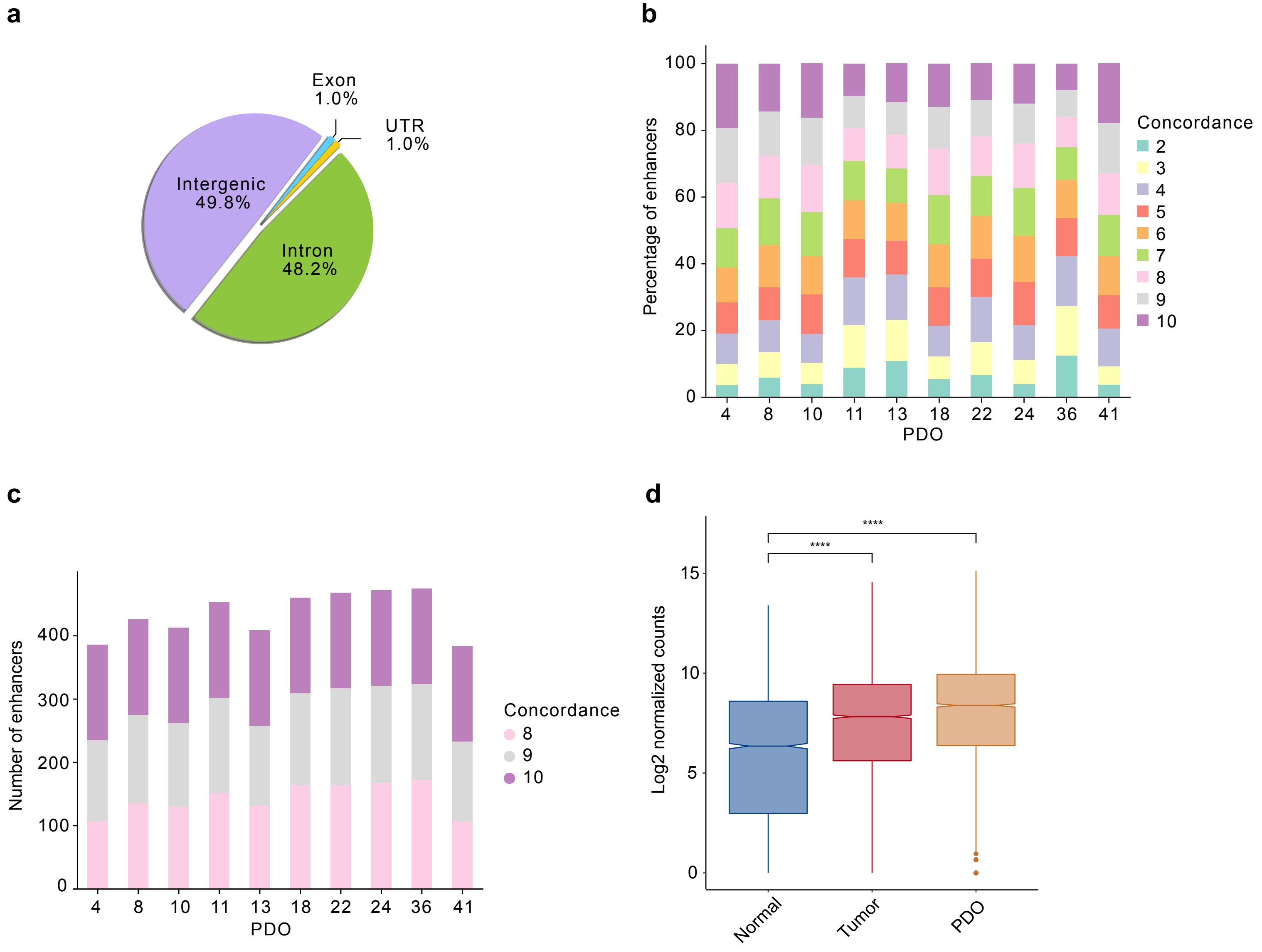

Supplementary Figure 4

The genomic localization of CRC gained enhancers and their distribution across PDOs

a, Pie chart showing the localization of the 2,419 ChromHMM-defined gained enhancers within functional features of the genome. UTR, untranslated region.

b, Percentage of concordant gained enhancers across patient-derived organoids (PDOs).

c, Number of conserved gained enhancers across PDOs.

d, Boxplots of RNA-seq log2 normalized counts showing the expression distribution of genes that are annotated to gained active enhancers and upregulated in PDOs (n = 495) across normal colon tissues (n = 9), primary tumors (n = 10), and PDOs (n = 10). Boxplots describe the median (middle line) and interquartile range (box denoting first and third percentile) with whiskers denoting the minimum and maximum within the 1.5 x interquartile range and outlying points beyond the whiskers plotted individually. ****$P < 0.0001$, two-sided Wilcoxon rank sum exact test.

Supplementary Figure 5

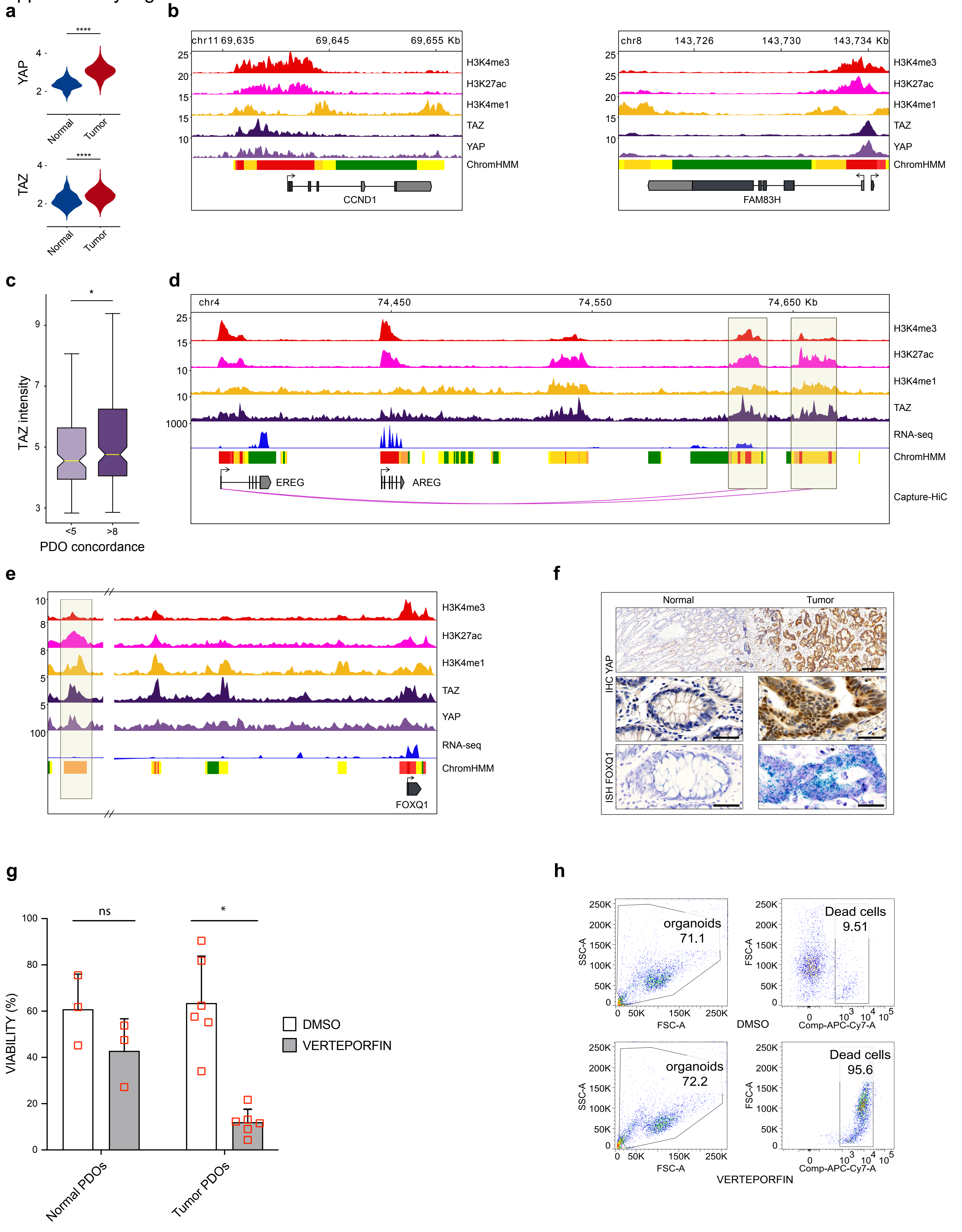

Supplementary Figure 5

Genomic overview and gene expression of YAP/TAZ target genes

a, *YAP* and *TAZ* are transcriptionally upregulated in primary tumors (n = 456) compared to normal colon tissues (n = 41) in the TCGA colon adenocarcinoma dataset. Violin plots of log2 normalized gene counts adjusted for epithelial cell frequency (Methods). ****P < 0.0001, two-sided Wilcoxon rank sum exact test.

b, Genomic overview of YAP/TAZ canonical targets, *CCND1* (left) and *FAM83H* (right), showing H3K4me3, H3K27ac, H3K4me1, TAZ, and YAP profiles and ChromHMM states. See Fig. 2c,e for details on ChromHMM tracks.

c, TAZ signal intensity is higher in conserved (shared by 8-10 PDOs; n = 195) compared to non-conserved (shared by 1-5 PDOs; n = 149) gained enhancers. Boxplots describe the median (middle line) and interquartile range (box denoting first and third percentile) with whiskers denoting the minimum and maximum within the 1.5 x interquartile range. *P = 0.028, two-sided Mann Whitney U test exact *P*-value. PDO, patient-derived organoid.

d, Representative genomic overview of *EREG*, a YAP/TAZ target gene. The tracks show H3K4me3, H3K27ac, H3K4me1 and TAZ profiles, RNA-seq signals, ChromHMM states, and capture Hi-C promoter-enhancer interactions.

e, Genomic overview of *FOXQ1* (hg38, chr6: 1,049,299 - 1,319,692) showing H3K4me3, H3K27ac, H3K4me1, TAZ, and YAP profiles, RNA-seq signals and ChromHMM states.

f, *FOXQ1* expression in the same tissues expressing YAP. Images of a human CRC (right) and nearby healthy colon mucosa (left) tissue within the same section. The graphs show immunohistochemical (IHC) staining for YAP (upper and middle panels; scale bars, 250 and 50 mm, respectively) and RNA in situ hybridization (ISH) for *FOXQ1* (bottom panel; scale bars, 50 mm). Nuclei were counterstained with hematoxylin.

g, Cell viability in normal and tumor organoids treated with DMSO (control) or 1 μM of verteporfin and cultured for 48 hours. Tumor organoids show a significant reduction in viable

cells compared to DMSO-treated cells (mean ± s.d., n: Normal = 3 and Tumor = 6 independent CRC patients). **P* = 0.016, one-sided Wilcoxon signed rank test exact *P*-value.

h, Gating strategy for assessing PDO cell viability (Fig. 4j and Supplementary Fig. 5g).

Supplementary Figure 6

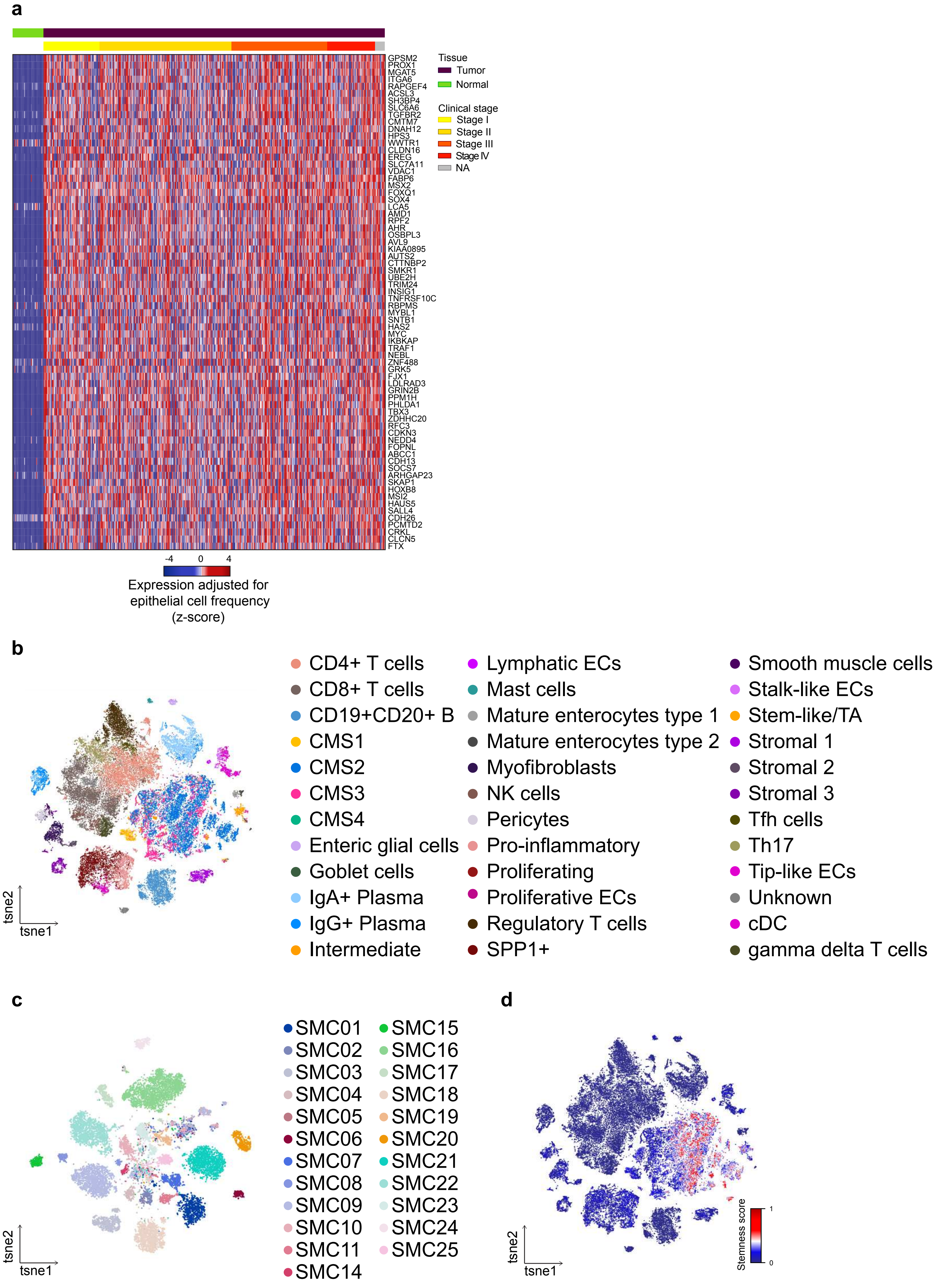

Supplementary Figure 6

Expression of YAP/TAZ-controlled genes and characterization of cell populations in 23 CRC patients

a, Heatmap of RNA-seq log2 normalized counts for target genes of YAP/TAZ-controlled enhancers in the TCGA COAD dataset. Tissue populations and clinical stages are represented by color-coded bars above the heatmap. Expression values of TCGA bulk RNA-seq data were adjusted for epithelial cell frequency (see Methods for details).

b, Detailed t-SNE visualization of all cell populations identified in the dataset. Cell type designations are based on the original clustering[2].

c, Sub-clustering analysis of 18,539 tumor and normal epithelial cells showing patient-specific clustering of most tumor cells. d, t-SNE plot depicting the distribution of the stemness score across all cell populations.

Supplementary Table 1 | Aggregated clinical information of patients

Age	Sex	Location	Histology	Grade	pT	pN	Microsatellite status
Average = 76	F = 8	A = 7	mod = 5	G2 = 7	pT2 = 4	N0 = 6	MSI = 2
	M= 3	T = 1	muc = 1	G3 = 4	pT3 = 5	N1a = 1	MSS = 9
		S = 2	ulc = 2		pT4 = 1	N1b = 3	
		R = 1	angio = 1		pT4a = 1	N2a = 1	
			na = 2				

Sex: F, female; M, male

Location: A, ascending colon; T, transversal colon; S, sigmoid colon; R, rectum

Histology: mod, moderately differentiated adenocarcinoma; muc, mucinous adenocarcinoma; ulc, ulcerated; angio, angioinvasive; na, not available

Microsatellite status: MSI, Microsatellite instable; MSS, Microsatellite stable

Supplementary Table 2 | Antibodies used in the study

Antibody	Working dilution / Concentration	Company	Catalogue No.	Assay
Rabbit polyclonal anti-EpCAM	1:50	R&D Systems	AF960	IF
Rabbit monoclonal anti-Ki67	1:250	Abcam	92742 - EPR3610	IF
Rabbit polyclonal anti-Fabp1	1:250	SA	HPA028275	IF
Rabbit polyclonalanti-Cytokeratin 20	1:100	Abcam	97511	IF
Mouse monoclonal anti-Mucin2	1:50	SCB	sc-515032 - F-2	IF
Mouse monoclonal anti-Chromogranin A	1:50	SCB	sc-393941- H-300	IF
Rabbit polyclonal anti-LAMA5	1:50	SA	SAB4501720	IF
Alexa Fluor 647 Phalloidin	1:50	TFS	A22287	IF
Alexa Fluor 568 Phalloidin	1:50	TFS	A12380	IF
Alexa Fluor 488 Goat anti mouse	1:500	TFS	A11029	IF
Alexa Fluor 488 Donkey anti goat	1:500	TFS	A11055	IF
Alexa Fluor 568 Donkey anti rabbit	1:500	TFS	A10042	IF
Alexa Fluor 647 Donkey anti rabbit	1:500	TFS	A31573	IF
Rabbit polyclonal anti-Histone H3 (tri methyl Lys4)	1 µg	Millipore	07-473	ChIP
Rabbit polyclonal anti-Histone H3 (mono methyl Lys4)	1.5 µg	Diagenode	C15410194	ChIP
Rabbit polyclonal anti-Histone H3 (acetyl Lys27)	1 µg	Abcam	4729	ChIP
Rabbit polyclonal anti-Histone H3 (tri methyl Lys36)	1.6 µg	Diagenode	C15410192	ChIP
Rabbit polyclonal anti-Histone H3 (tri methyl Lys27)	1 µg	Millipore	07-449	ChIP
Rabbit polyclonal anti-TAZ (WWTR1)	0.4 µg	SA	HPA007415	ChIP
Rabbit monoclonal anti-YAP1	0.4 µg	Abcam	52771 - EP1674Y	ChIP
normal rabbit control IgG		SB	CR1	ChIP
Rabbit anti WWTR1/TAZ	1:20	SA	HPA007415	IHC
Mouse monoclonal anti YAP1	1:100	SCB	Sc-101199	IHC
Fixable Viability Stain 780 (FVS780)	1:1000	BD HORIZON™	565388	FACS

Company: TFS, Thermo Fisher Scientific; SCB, Santa Cruz Biotechnology; SA, Sigma Aldrich; SB, Sino Biological
Assay: IF, Immunofluorescence; ChIP, Chromatin Immunoprecipitation; IHC, Immunohistochemistry

Supplementary Table 3 | Nextera primers for ChIPmentation library preparation[3]

Index 1 (i5)		
**Sample**	**Index 1 ID (i5)**	**Index 1 sequence (Tag included in forward read)**
SQ: 2353	Ad1.3_TATCCTCT	AATGATACGGCGACCACCGAGATCTACAC**TATCCTCT**TCGTCGGCAGCGTCAGATGTGTAT
SQ_2354	Ad1.3_TATCCTCT	AATGATACGGCGACCACCGAGATCTACAC**TATCCTCT**TCGTCGGCAGCGTCAGATGTGTAT
SQ_2355	Ad1.7_AAGGAGTA	AATGATACGGCGACCACCGAGATCTACAC**AAGGAGTA**TCGTCGGCAGCGTCAGATGTGTAT
SQ_2357	Ad1.7_AAGGAGTA	AATGATACGGCGACCACCGAGATCTACAC**AAGGAGTA**TCGTCGGCAGCGTCAGATGTGTAT
SQ_2358	Ad1.7_AAGGAGTA	AATGATACGGCGACCACCGAGATCTACAC**AAGGAGTA**TCGTCGGCAGCGTCAGATGTGTAT
SQ_2359	Ad1.7_AAGGAGTA	AATGATACGGCGACCACCGAGATCTACAC**AAGGAGTA**TCGTCGGCAGCGTCAGATGTGTAT
SQ_2360	Ad1.7_AAGGAGTA	AATGATACGGCGACCACCGAGATCTACAC**AAGGAGTA**TCGTCGGCAGCGTCAGATGTGTAT
SQ_2361	Ad1.7_AAGGAGTA	AATGATACGGCGACCACCGAGATCTACAC**AAGGAGTA**TCGTCGGCAGCGTCAGATGTGTAT
SQ_2362	Ad1.7_AAGGAGTA	AATGATACGGCGACCACCGAGATCTACAC**AAGGAGTA**TCGTCGGCAGCGTCAGATGTGTAT
**Index 2 (i7)**		
**Sample**	**Index 2 ID (i7)**	**Index 2 sequence (Tag included in reverse read)**
SQ_2353	Ad2.10_CGAGGCTG	CAAGCAGAAGACGGCATACGAGAT**CAGCCTCG**GTCTCGTGGGCTCGGAGATGTG
SQ_2354	Ad2.12_GTAGAGGA	CAAGCAGAAGACGGCATACGAGAT**TCCTCTAC**GTCTCGTGGGCTCGGAGATGTG
SQ_2355	Ad2.1_TAAGGCGA	CAAGCAGAAGACGGCATACGAGAT**TCGCCTTA**GTCTCGTGGGCTCGGAGATGTG
SQ_2357	Ad2.5_GGACTCCT	CAAGCAGAAGACGGCATACGAGAT**AGGAGTCC**GTCTCGTGGGCTCGGAGATGTG
SQ_2358	Ad2.10_CGAGGCTG	CAAGCAGAAGACGGCATACGAGAT**CAGCCTCG**GTCTCGTGGGCTCGGAGATGTG
SQ_2359	Ad2.4_TCCTGAGC	CAAGCAGAAGACGGCATACGAGAT**GCTCAGGA**GTCTCGTGGGCTCGGAGATGTG
SQ_2360	Ad2.7_CTCTCTAC	CAAGCAGAAGACGGCATACGAGAT**GTAGAGAG**GTCTCGTGGGCTCGGAGATGTG
SQ_2361	Ad2.8_CAGAGAGG	CAAGCAGAAGACGGCATACGAGAT**CCTCTCTG**GTCTCGTGGGCTCGGAGATGTG
SQ_2362	Ad2.12_GTAGAGGA	CAAGCAGAAGACGGCATACGAGAT**TCCTCTAC**GTCTCGTGGGCTCGGAGATGTG

The index within each sequence is shown in red

Supplementary References

1. Isella, C. *et al.* Stromal contribution to the colorectal cancer transcriptome. *Nat Genet* **47**, 312-319, doi:10.1038/ng.3224 (2015).
2. Lee, H. O. et al. Lineage-dependent gene expression programs influence the immune landscape of colorectal cancer. *Nat Genet* **52**, 594-603, doi:10.1038/s41588-020-0636-z (2020).
3. Schmidl, C., Rendeiro, A. F., Sheffield, N. C. & Bock, C. ChIPmentation: fast, robust, low-input ChIP-seq for histones and transcription factors. *Nat Methods* **12**, 963-965, doi:10.1038/nmeth.3542 (2015).
