# Supplementary material for: Epigenomic landscape of human colorectal cancer unveils an aberrant core of pan-cancer enhancers orchestrated by YAP/TAZ
Source: Nat Commun. 2021 Apr 20;12:2340. doi: 10.1038/s41467-021-22544-y (PMC8058065; doi:10.1038/s41467-021-22544-y)
Supplement: Supplementary file 3 — Description of Additional Supplementary Files [file 41467_2021_22544_MOESM3_ESM.pdf]

Description of Additional Supplementary Files

Supplementary Data 1. List of RNA-seq and ChIP-seq samples used in the study.

Supplementary Data 2. Highly variable immune- and stromal-related genes in CRC samples.

Supplementary Data 3. Details of ChIP-seq samples used for ChromHMM analysis.

Supplementary Data 4. Active distal ChromHMM-defined enhancer regions.

Supplementary Data 5. Motif discovery data for tumor-specific conserved gained enhancers. Motif enrichment in target versus background sequences was performed using the default cumulative binomial distribution as scoring function with correction for multiple testing (q-values; Benjamini).

Supplementary Data 6. Gene annotation of tumor-specific gained enhancers.

Supplementary Data 7. Peak calling of TAZ ChIP-seq data. A poisson CDF (cumulative distribution function) is applied to calculate one-side P-values for enrichment with false discovery rate correction (qValue).

Supplementary Data 8. Highly conserved gained enhancer regions, YAP/TAZ-targeted and associated to TCGA COAD ATAC-seq data.

Supplementary Data 9. Public H3K27ac ChIP-seq samples of primary tumors and normal tissues used in the analysis of pan-cancer regions.

Supplementary Data 10. Gene lists used for combined score analysis.
