# Supplementary material for: Epigenomic landscape of human colorectal cancer unveils an aberrant core of pan-cancer enhancers orchestrated by YAP/TAZ
Source: Nat Commun. 2021 Apr 20;12:2340. doi: 10.1038/s41467-021-22544-y (PMC8058065; doi:10.1038/s41467-021-22544-y)
Supplement: Supplementary file 14 — Reporting Summary [file 41467_2021_22544_MOESM14_ESM.pdf]

nature research

Corresponding author(s): Massimiliano Pagani

Last updated by author(s): Feb 26, 2021

Reporting Summary

Nature Research wishes to improve the reproducibility of the work that we publish. This form provides structure for consistency and transparency in reporting. For further information on Nature Research policies, see our Editorial Policies and the Editorial Policy Checklist.

Statistics

For all statistical analyses, confirm that the following items are present in the figure legend, table legend, main text, or Methods section.

n/a	Confirmed	
☐	☒	The exact sample size (*n*) for each experimental group/condition, given as a discrete number and unit of measurement
☐	☒	A statement on whether measurements were taken from distinct samples or whether the same sample was measured repeatedly
☐	☒	The statistical test(s) used AND whether they are one- or two-sided<br>*Only common tests should be described solely by name; describe more complex techniques in the Methods section.*
☒	☐	A description of all covariates tested
☐	☒	A description of any assumptions or corrections, such as tests of normality and adjustment for multiple comparisons
☐	☒	A full description of the statistical parameters including central tendency (e.g. means) or other basic estimates (e.g. regression coefficient) AND variation (e.g. standard deviation) or associated estimates of uncertainty (e.g. confidence intervals)
☐	☒	For null hypothesis testing, the test statistic (e.g. *F*, *t*, *r*) with confidence intervals, effect sizes, degrees of freedom and *P* value noted<br>*Give P values as exact values whenever suitable.*
☒	☐	For Bayesian analysis, information on the choice of priors and Markov chain Monte Carlo settings
☒	☐	For hierarchical and complex designs, identification of the appropriate level for tests and full reporting of outcomes
☐	☒	Estimates of effect sizes (e.g. Cohen's *d*, Pearson's *r*), indicating how they were calculated

Our web collection on statistics for biologists contains articles on many of the points above.

Software and code

Policy information about availability of computer code

Data collection	RNA-seq libraries were constructed according to the TruSeq mRNA Stranded preparation kit (Illumina) and sequenced at HiSeq2500 with paired-end reads.<br>ChIP-seq libraries were constructed with TruSeq ChIP Library Preparation Kit (Illumina) and sequenced on Illumina HiSeq2500 platform with single-end reads.
Data analysis	Statistical analysis was performed in R (v3.5.1) and graphing in Illustrator (v25.0). Image analysis was performed using Fiji (v2.1.0/1.53c).<br>Quality control of RNA-seq data was performed with FastQC (v0.11.7) and MultiQC (v1.5). The reads were trimmed using BBDuk – BBMap v38.16 and aligned to the human hg38 reference using STAR (v2.5.3a). Quantification was performed using featureCounts – Subread v1.6.2 with default parameters. Normalization and differential analysis were carried out using DESeq2 package (v1.22.2) and R (v3.5.1).<br>Quality control of ChIP-seq data was performed with FastQC (v0.11.7.1). The reads were aligned to the human hg38 reference genome (GENCODE Release 25 basic gene annotation) using Bowtie (v1.2.2), sorted using SAMtools (v1.8) and directly converted into binary files (BAM). PCR duplicated reads were marked and removed using SAMtools (v1.8). The peaks were called with MACS2 (v2.1.0) using matched input DNA as a control and appropriate options for sharp and broad histone modifications. Peaks overlapping ENCODE blacklisted regions, found in un-placed and un-localized scaffolds were removed. Normalized coverage tracks were generated using the bamCoverage function from deepTools suite (v2.5.7). A consensus peakset was generated using DiffBind (v2.6.6). De novo chromatin state characterization was performed in ChromHMM (v1.12). Motif binding discovery was performed using HOMER (v4.7). Analysis of public single-cell data was done using the python package Scanpy (v1.4.2).<br>RNA-seq and ChIP-seq primary analyses were executed by a custom pipeline managed by Nextflow (v20.04.1.5335). The code used in this study has been deposited on GitHub (https://github.com/paganilab/DellaChiara_et_al_2021).

For manuscripts utilizing custom algorithms or software that are central to the research but not yet described in published literature, software must be made available to editors and reviewers. We strongly encourage code deposition in a community repository (e.g. GitHub). See the Nature Research guidelines for submitting code & software for further information.

Data

Policy information about availability of data

All manuscripts must include a data availability statement. This statement should provide the following information, where applicable:

- Accession codes, unique identifiers, or web links for publicly available datasets
- A list of figures that have associated raw data
- A description of any restrictions on data availability

The RNA-seq and ChIP-seq data generated during this study are available at the European Nucleotide Archive with accession numbers E-MTAB-8448 [https://www.ebi.ac.uk/arrayexpress/experiments/E-MTAB-8448/] and E-MTAB-8416 [https://www.ebi.ac.uk/arrayexpress/experiments/E-MTAB-8416/], respectively. The epigenomic (ChIP-seq on histone marks and ChromHMM tracks) and transcriptomic (RNA-seq) data for all the PDOs analyzed in this study are publicly available at the HePIC web browser at https://ifom.eu/bioinformatics/hepic. Source data are provided with this paper. The remaining data are available within the Article, Supplementary Information or available from the authors upon request.

Publicly available datasets used in this study can be found under accession codes GSE77737 [https://www.ncbi.nlm.nih.gov/geo/query/acc.cgi?acc= GSE77737] and GSE132465 [https://www.ncbi.nlm.nih.gov/geo/query/acc.cgi?acc=GSE132465] (colon), GSE51776 [https://www.ncbi.nlm.nih.gov/geo/query/acc.cgi?acc=GSE51776] (gastric cancer), GSE114737 [https://www.ncbi.nlm.nih.gov/geo/query/acc.cgi?acc=GSE114737] (breast and endometrial cancer), GSE74230 [https://www.ncbi.nlm.nih.gov/geo/query/acc.cgi?acc=GSE74230] (osteosarcoma), GSE101065 [https://www.ncbi.nlm.nih.gov/geo/query/acc.cgi?acc=GSE101065] and GSE142924 [https://www.ncbi.nlm.nih.gov/geo/query/acc.cgi?acc=GSE142924] (uterus), GSE16256 [https://www.ncbi.nlm.nih.gov/geo/query/acc.cgi?acc=GSE16256] (liver, adrenal gland, pancreas), GSE96504 [https://www.ncbi.nlm.nih.gov/geo/query/acc.cgi?acc=GSE96504] (liver), GSE101019 [https://www.ncbi.nlm.nih.gov/geo/query/acc.cgi?acc=GSE101019] and GSE95966 [https://www.ncbi.nlm.nih.gov/geo/query/acc.cgi?acc=GSE95966] (adrenal gland), GSE101258 [https://www.ncbi.nlm.nih.gov/geo/query/acc.cgi?acc=GSE101258], GSE96258 [https://www.ncbi.nlm.nih.gov/geo/query/acc.cgi?acc=GSE96258], GSE95981 [https://www.ncbi.nlm.nih.gov/geo/query/acc.cgi?acc=GSE95981], and GSE142995 [https://www.ncbi.nlm.nih.gov/geo/query/acc.cgi?acc=GSE142995] (thyroid gland), GSE101269 [https://www.ncbi.nlm.nih.gov/geo/query/acc.cgi?acc=GSE101269], GSE101231 [https://www.ncbi.nlm.nih.gov/geo/query/acc.cgi?acc=GSE101231], GSE142968 [https://www.ncbi.nlm.nih.gov/geo/query/acc.cgi?acc=GSE142968], GSE96212 [https://www.ncbi.nlm.nih.gov/geo/query/acc.cgi?acc=GSE96212] (pancreas).

Field-specific reporting

Please select the one below that is the best fit for your research. If you are not sure, read the appropriate sections before making your selection.

[x] Life sciences [ ] Behavioural & social sciences [ ] Ecological, evolutionary & environmental sciences

For a reference copy of the document with all sections, see nature.com/documents/nr-reporting-summary-flat.pdf

Life sciences study design

All studies must disclose on these points even when the disclosure is negative.

Sample size	No statistical methods were used to determine the sample size. We used all the samples available in each dataset.
Data exclusions	No replicates were excluded from analyses.
Replication	Experiments were performed at least three times with reproducible results obtained. Images are representative of experiments repeated at least three times under similar conditions.
Randomization	No randomization was used. Samples were allocated into experimental groups based on prior knowledge of the clinical features (surgical resection of primary colonic normal or tumoral tissue). Patient-derived organoids were derived from primary tumors that were representative of the genetic and molecular heterogeneity in colorectal cancer (CRC) based on three gene-expression classification systems and the MSI/MSS classification. Since our aim is to assess the shared epigenetic regulation of molecularly diverse CRCs, control of covariates is not relevant in our study.
Blinding	No blinding was performed during data collection and processing since the morphological characteristics of the samples, normal and tumoral tissues were visibly different. All patient-derived organoids were tumoral thus blinding was not relevant.

Reporting for specific materials, systems and methods

We require information from authors about some types of materials, experimental systems and methods used in many studies. Here, indicate whether each material, system or method listed is relevant to your study. If you are not sure if a list item applies to your research, read the appropriate section before selecting a response.

Materials & experimental systems

n/a	Involved in the study	
☐	☒	Antibodies
☒	☐	Eukaryotic cell lines
☒	☐	Palaeontology and archaeology
☒	☐	Animals and other organisms
☐	☒	Human research participants
☒	☐	Clinical data
☒	☐	Dual use research of concern

Methods

n/a	Involved in the study	
☐	☒	ChIP-seq
☐	☒	Flow cytometry
☒	☐	MRI-based neuroimaging

Antibodies

Antibodies used

Rabbit polyclonal anti-EpCAM, R&D Systems AF960
Rabbit monoclonal anti-Ki67, Abcam 92742 - EPR3610
Rabbit polyclonal anti-Fabp1, Sigma Aldrich HPA028275
Rabbit polyclonalanti-Cytokeratin 20, Abcam 97511
Mouse monoclonal anti-Mucin2, Santa Cruz Biotechnology sc-515032 - F-2
Mouse monoclonal anti-Chromogranin A, Santa Cruz Biotechnology sc-393941- H-300
Rabbit polyclonal anti-LAMA5, Sigma Aldrich SAB4501720
Alexa Fluor 647 Phalloidin, Thermo Fisher Scientific A22287
Alexa Fluor 568 Phalloidin, Thermo Fisher Scientific A12380
Alexa Fluor 488 Goat anti mouse, Thermo Fisher Scientific A11029
Alexa Fluor 488 Donkey anti goat, Thermo Fisher Scientific A11055
Alexa Fluor 568 Donkey anti rabbit Thermo Fisher Scientific A10042
Alexa Fluor 647 Donkey anti rabbit, Thermo Fisher Scientific A31573
Rabbit polyclonal anti-Histone H3 (tri methyl Lys4) Millipore 07-473
Rabbit polyclonal anti-Histone H3 (mono methyl Lys4) DIAGENODE C15410194
Rabbit polyclonal anti-Histone H3 (acetyl Lys27) Abcam 4729
Rabbit polyclonal anti-Histone H3 (tri methyl Lys36) DIAGENODE C15410192
Rabbit polyclonal anti-Histone H3 (tri methyl Lys27) Millipore 07-449
Rabbit polyclonal anti-TAZ (WWTR1) Sigma Aldrich HPA007415
Rabbit monoclonal anti-YAP1 (YAP) Abcam 52771
normal rabbit control IgG Sino Biological CR1
anti-YAP, Santa Cruz Biotechnology – Sc-101199
Fixable Viability Stain 780 (FVS780) BD HORIZON, 565388

Validation

All antibodies used in the study are commercially available (R&D Systems, Millipore, Diagenode, Abcam, Sigma Aldrich, Thermo Fisher Scientific, Sino Biological, Santa Cruz Biotechnology, BD HORIZON) and were titrated in-house as indicated in the main manuscript and Supplementary Information. Validation statements and details are provided by the manufacturer:

Rabbit monoclonal anti-Ki67 Abcam 92742 - EPR3610
Rabbit polyclonalanti-Cytokeratin 20 Abcam 97511
Rabbit polyclonal anti-Histone H3 (acetyl Lys27) Abcam 4729
Rabbit monoclonal anti-YAP1 EP1674Y Abcam 52771
https://www.abcam.com/primary-antibodies/a-guide-to-antibody-validation

Rabbit polyclonal anti-EpCAM R&D Systems AF960
https://www.rndsystems.com/tags/antibody-validation

Rabbit polyclonal anti-Histone H3 (mono methyl Lys4) DIAGENODE C15410194
Rabbit polyclonal anti-Histone H3 (tri methyl Lys36) DIAGENODE C15410192
https://www.diagenode.com/en/applications/chip-seq-antibodies

Mouse monoclonal anti-Mucin2 Santa Cruz Biotechnology sc-515032 - F-2
Mouse monoclonal anti-Chromogranin A Santa Cruz Biotechnology sc-393941- H-300
https://www.scbt.com/resources/protocols/immunofluorescence-cell-staining

normal rabbit control IgG Sino Biological CR1
https://www.sinobiological.com/category/ip-validated-by-multiple-cells

Human research participants

Policy information about studies involving human research participants

Population characteristics	Colorectal cancer organoids derived from 10 patients (3 males: 7 females, average age 76 years old).
Recruitment	The study involves volunteer colorectal cancer patients for which surgically resected specimens of primary tumors were obtained.
Ethics oversight	The Institutional Review Boards of San Gerardo Hospital (Department of Surgery), Monza and UO Chirurgia Epatobiliopancreatica e Digestiva Ospedale San Paolo, Milan provided ethical approval of the study.

Note that full information on the approval of the study protocol must also be provided in the manuscript.

ChIP-seq

Data deposition

- [x] Confirm that both raw and final processed data have been deposited in a public database such as GEO.
- [x] Confirm that you have deposited or provided access to graph files (e.g. BED files) for the called peaks.

Data access links<br>*May remain private before publication.*	The RNA-seq and ChIP-seq data generated during this study are available at the European Nucleotide Archive with accession numbers E-MTAB-8448 [https://www.ebi.ac.uk/arrayexpress/experiments/E-MTAB-8448/] and E-MTAB-8416 [https://www.ebi.ac.uk/arrayexpress/experiments/E-MTAB-8416/] and at the web browser https://ifom.eu/bioinformatics/hepic.
Files in database submission	https://www.ebi.ac.uk/arrayexpress/files/E-MTAB-8448/E-MTAB-8448.sdrf.txt<br>https://www.ebi.ac.uk/arrayexpress/files/E-MTAB-8416/E-MTAB-8416.sdrf.txt
Genome browser session<br>(e.g. UCSC)	https://ifom.eu/bioinformatics/hepic

Methodology

Replicates	10 Colorectal cancer patient-derived organoids.
Sequencing depth	ChIP-seq data was single-end with a median depth of 45 million reads per sample.
Antibodies	Rabbit polyclonal anti-Histone H3 (tri methyl Lys4) (Millipore, 07-473); Rabbit polyclonal anti-Histone H3 (mono methyl Lys4) (DIAGENODE, C15410194); Rabbit polyclonal anti-Histone H3 (acetyl Lys27) (Abcam, 4729); Rabbit polyclonal anti-Histone H3 (tri methyl Lys36) (DIAGENODE C15410192); Rabbit polyclonal anti-Histone H3 (tri methyl Lys27) (Millipore,07-449); Rabbit polyclonal anti-TAZ (WWTR1) (Sigma Aldrich, HPA007415); Rabbit monoclonal anti-YAP1 (YAP) (Abcam, 52771); normal rabbit control IgG (Sino Biological, CR1)
Peak calling parameters	The peaks were called with MACS2 (v2.1.0) (command line parameters: --nomodel --ext; size 200 -B -q 0.01 for sharp histone modifications H3K4me3 and H3K27Ac, and adding --broad for broad histone modifications H3K4me1, H3K36me3 and H3K27me3) using matched input DNA as a control. Peaks overlapping ENCODE blacklisted regions hg38 (i.e. regions in the human genome with signal artefacts in next generation sequencing experiments, (https://www.encodeproject.org/annotations/ENCSR636HFF/) were removed. Peaks found in un-placed and un-localized scaffolds were removed.
Data quality	Quality control of the reads was performed with FastQC v0.11.7, MultiQC v1.5, hierarchical clustering and principal component analysis.
Software	ChIP-seq primary analyses were executed by a custom pipeline managed by Nextflow. Quality control of ChIP-seq data was performed with FastQC v0.11.7.1. The reads were aligned to the human hg38 reference genome (GENCODE Release 25 basic gene annotation) using bowtiev1.2.2, sorted using SAMtoolsv1.8 and directly converted into binary files (BAM). PCR duplicated reads were marked and removed using SAMtoolsv1.8. The peaks were called with MACS2v2.1.0.

Flow Cytometry

Plots

Confirm that:

- [x] The axis labels state the marker and fluorochrome used (e.g. CD4-FITC).
- [x] The axis scales are clearly visible. Include numbers along axes only for bottom left plot of group (a 'group' is an analysis of identical markers).
- [x] All plots are contour plots with outliers or pseudocolor plots.
- [x] A numerical value for number of cells or percentage (with statistics) is provided.

Methodology

Sample preparation	Samples used for flow cytometry were normal and tumoral PDOs. They were treated for 48h with 1mM VERTEPORFIN or DMSO and then dissociated into single cells by using TryPLE express for 5 min at 37oC. Cells were stained with Fixable Viability Stain 780 (FVS780 - BD HORIZONTM,565388) according to manufacturer's instructions.
Instrument	Samples were acquired at FACS Canto II (Becton Dickinson).
Software	All FACS data were analysed using FlowJo 10.7.1.
Cell population abundance	A minimum of 5,000 cells/sample were acquired for viability staining.
Gating strategy	The gating strategy was done as exemplified in Supplementary Fig. 5h: FSC/SSC > cells of interest > FSC/APCCy7 > live/dead staining.

☒ Tick this box to confirm that a figure exemplifying the gating strategy is provided in the Supplementary Information.
